# Creation of Ionically Crosslinked Tri-Layered Chitosan Membranes to Simulate Different Human Skin Properties

**DOI:** 10.3390/ma14071807

**Published:** 2021-04-06

**Authors:** Rocío Guerle-Cavero, Blanca Lleal-Fontàs, Albert Balfagón-Costa

**Affiliations:** Pharmaceutical Chemistry Research Group, Instituto Químico de Sarriá, 08017 Barcelona, Spain; blancallealf@iqs.url.edu (B.L.-F.); albert.balfagon@iqs.url.edu (A.B.-C.)

**Keywords:** chitosan, crosslinking, membrane, pores, layers, elasticity, permeation, human skin, ethics

## Abstract

In 2023, new legislation will ban the use of animals in the cosmetic industry worldwide. This fact, together with ethical considerations concerning the use of animals or humans in scientific research, highlights the need to propose new alternatives for replacing their use. The aim of this study is to create a tri-layered chitosan membrane ionically crosslinked with sodium tripolyphosphate (TPP) in order to simulate the number of layers in human skin. The current article highlights the creation of a membrane where pores were induced by a novel method. Swelling index, pore creation, and mechanical property measurements revealed that the swelling index of chitosan membranes decreased and, their pore formation and elasticity increased with an increase in the Deacetylation Grade (DDA). Additionally, the results demonstrate that chitosan’s origin can influence the elastic modulus value and reproducibility, with higher values being obtained with seashell than snow crab or shrimp shells. Furthermore, the data show that the addition of each layer, until reaching three layers, increases the elastic modulus. Moreover, if layers are crosslinked, the elastic modulus increases to a much greater extent. The characterization of three kinds of chitosan membranes was performed to find the most suitable material for studying different human skin properties.

## 1. Introduction

Animal models are widely used in basic research as an alternative to human skin and in the preclinical studies phase for evaluating the action of drugs. Nonetheless, animal models rarely predict human responses in terms of skin physiology. Additionally, the high cost and time-consuming protocols are important drawbacks of their use [1]. Since 2013, ethical considerations regarding the use of animals and/or volunteers for scientific research have been reinforced in the European Union [2]. Specifically, in dermatology, regulatory authorities and industry are demanding artificial skin substitutes since the use of animals and humans is ethically regulated for drugs and forbidden for cosmetic use. In 2018, the EU Parliament encouraged a worldwide ban on testing cosmetics on animals by 2023 [3,4].

This fact, together with the lack of reproducibility of results when using different human skin, has encouraged scientists to propose new and reproducible alternatives for proving the safety and efficacy of topical products and ingredients. Until now, there have been different proposals of skin-like substitutes in an attempt to reproduce several aspects of real human skin, but, for many reasons, they are not considered as perfect human skin alternatives [1]. In most cases, living cells are cultured in an artificial scaffold, which means that the skin is difficult to manage and affects its costs and expiry date [5,6].

The purpose of this research is to create a physical skin model based on a biomaterial scaffold, which can prove similar results to human skin but with strong simplifications that do not reflect the structure and the composition of the human skin [7]. This physical model consists of a tri-layered membrane composed of cost-effective materials which can mimic several skin characteristics and properties such as layers, skin pore size, elasticity, and hydration, avoiding the use of living cells, and with no aim to reproduce the human skin condition and its complexity. The scaffold of the membrane is mainly composed of chitosan crosslinked with TPP.

The main reasons that lead us to opt for chitosan, instead of other biomaterials naturally found in skin as the scaffold of these membranes, are:

1. It is a sustainable ingredient, as it is a waste product obtained from different animals’ seashells, in the case of our research.
2. Chitin, the biomaterial which chitosan is derived from, is the second most abundant natural polymer present in the world, after cellulose.
3. Chitosan is cost affordable compared to other biomaterials, which can be naturally found in skin such as collagen and elastin fibers, glycosaminoglycans such as hyaluronic acid, among others.
4. Chitosan is widely used in artificial skin models as reported in different papers [8].
5. We wanted to study the effect of different deacetylation grades (DDA) and the origin of chitosan ionically crosslinked to form three layers, but with no interaction of other biomaterials. Our aim was to choose the most suitable chitosan for studying different kinds of skin properties, before combining it with other biomaterials.
6. Chitosan is characterized by primary amines along the backbone. This structure allows particular interactions with proteins, cells and living organisms. Natural biomaterials, such as collagen and chitosan, are either protein or polysaccharide in nature. Their close resemblance with the natural Extracellular Matrix (ECM) makes them highly biocompatible and easy to degrade. Hence, either collagen or chitosan biomaterial-based scaffolds could be suitable for skin cell growth [9]. However, according to Ma et al. [10], during dermal fibroblast culture of 4 weeks, chitosan scaffolds did not contract compared to collagen scaffolds. Therefore, they suggested chitosan scaffolds as a good alternative to collagen-based artificial skin models.
7. To end with, some other possible applications of this chitosan-based membrane could be for biomedical applications. Chitosan has a wide range of applications in burn and wound treatments due to its hemostatic, antimicrobial and antifungal properties.

The membranes created in this research could be considered as physical skin models, based on the review by Dąbrowska, A. et al. [7]. The authors mentioned a wide list of materials for simulating some skin properties, which are not found in skin such as: agar, agarose, polyvinyl alcohol gels, elastomers, epoxy resins, metal and textiles between others.

It has previously been reported that material blending can result in different functional properties and improve the initial properties of the material. Different scientific papers have reported a combination of chitosan and sodium chondroitin, collagen, agarose, and hyaluronic acid, among others, so as to improve properties such as the elasticity, water uptake capacity, elongation at break, etc [8,9,10,11,12,13,14,15,16].

In the last ten years, different fabrication methods for the construction of three-dimensional scaffolds have been developed for tissue engineering and regenerative medicine, including electrospinning, phase-separation, freeze drying, and self-assembly [17].

The freeze drying technique, based on the principle of sublimation, is one of the most common methods used to create and control the internal pore size in chitosan scaffolds by modulating the freezing rate and temperature or pH. Firstly, the chitosan solution is frozen and, in a second stage, a lyophilization process is applied, consisting of removing the solvent under a high vacuum and low temperature. One major drawback of this technique is the long processing time required for the complete removal of solvent and the difficulty in precisely controlling the structure of scaffolds [1].

In this research, a polyelectrolite complex (PEC) was constructed by the well-known technique of Layer-by-Layer (LbL) self-assembly. Through this thin film fabrication technique, layers can be created in different ways, including immersive, spin, spray, fluidic, and electromagnetic assemblies, to name a few. PEC can provide unique mechanical, thermal, and physicochemical properties which individual components cannot [18,19]. In our case, a ladder-like structure was achieved. The application of characterization methods to monitor and evaluate LbL crosslinking is also discussed in this work [20,21,22].

Chitosan, as the main component of the membrane, is a hydrophilic polysaccharide copolymer composed of N-acetyl-D-glucose amine and D-glucose amine derived from chitin. The ratio of N-acetyl-D-glucose amine and D-glucose amine units determines the degree of deacetylation (DDA), which is a very important parameter affecting the final properties of chitosan. The DDA ranges from 50% up to 100%, with the latter representing fully deacetylated chitin [23].

Chitosan is a widely used biomaterial because of its unique characteristics, such as its reactivity, biocompatibility, biodegradability, non-toxicity, water absorption capacity, and renewal character, among others [24,25]. Many of these characteristics are due to the presence of primary amino groups (−NH_2_) in its structure. These groups are mainly formed by the deacetylation of chitin [26].

As has already been reported in previous studies, chitosan’s origin and deacetylation grade (DDA) can affect the physico-chemical properties of chitosan films [27,28]. In this project, three different kinds of chitosan were used in order to see how the origin and deacetylation grade (DDA) can influence three-layered membrane creation [29]. Two of them, with a DDA value of 85% and different origins, were obtained from a seashell skeleton and snow crab shell (*Chionoecetes opilio*). The third, with a DDA value of 76%, was obtained from shrimp shell (*Pandalus borealis*).

In this work, a tri-layered chitosan membrane was developed using chitosan-sodium tripolyphosphate ionically cross-linked [30,31,32,33,34].

Sodium tripolyphosphate (TPP) is used as an ionic crosslinker due to it being a nontoxic multivalent counterion and the fact that it can interact with chitosan and coagulate it. The existence of chitosan amino groups, with a positive charge, can lead to self-assembly with TPP that possesses a negative charge via ionic gelation [35]. TPP can form either intermolecular or intramolecular linkages with chitosan [36]. The ionotropic gelation method applied for the formation of multilayered cross-linked chitosan membranes can be easily performed by combining ionic cross-linking and deprotonation by adjusting the pH of either chitosan or TPP solution.

Crosslinking is a reaction where bonds are formed to hold portions of several polymer chains together. The more extensively cross-linked a polymer is, the more rigid and hard it is, in contrast to when the polymer is not cross-linked [37]. As has been demonstrated in this research, crosslinking between the different layers also brings more elasticity to the membrane, which reaches its maximum elasticity with three layers being crosslinked.

In order to achieve LbL crosslinking, first, chitosan is dissolved in an acid medium (acetic acid), in order to protonate its amine groups. Then, the solution is partially neutralized with sodium hydroxide, in order to neutralize part of those protonated amines. The reaction takes place when chitosan’s free amino groups are protonated (NH_3_^+^) to react with TTP negative charges (PO_3_^2−^). Thanks to the addition of sodium hydroxide at the beginning, a coiled conformation is avoided at the moment of crosslinking, so achieving a plain membrane is more feasible because the electrostatic interactions are not so strong [36].

The pH value of TPP on chitosan-tripolyphosphate cross-linking is also an essential parameter for the creation of a multi-layered membrane. When TPP is dissolved in water, it dissociates into Na^+^ and tripolyphosphate (P_3_O_10_^5−^) ions, but P_3_O_10_^5−^ ions undergo the process of hydrolysis. As a consequence, different forms of TPP ions can coexist in the solution including H_4_P_3_O_10_^−^, H_3_P_3_O_10_^2−^, H_2_P_3_O_10_^3−^, HP_3_O_10_^4−^, P_3_O_10_^5−^, and OH^−^, depending on the pH of the solution. Their pKa values are pK_1_ = 1, pK_2_ = 2, pK_3_ = 2.79, pK_4_ = 6.47 and pK_5_ = 9.24 of TPP and the pKa is around 6.5 for chitosan, with some slight variations depending on the DDA, being higher with a lower DDA [38,39,40].

When the pH of TPP is acidic, all TPP is crosslinked with NH_3_^+^ and there are no free TPP negative charges available for crosslinking of the next layer.

However, at a basic pH, both TPP ions and OH^–^ are present and may compete to interact with the –NH_3_^+^ of chitosan by ionic crosslinking and deprotonation, respectively [35,36]. In this research, TPP was dissolved in water (pH = 9), in order to allow the presence of free phosphoric ions due to OH^−^ competition, which could react with the −NH_3_^+^ charges of the next chitosan layer, enabling cross-linking between membrane layers. In this way, an ionic crosslinking reaction between layers was performed to complete the membrane.

Finally, in order to complete the crosslinking and get the best membrane performances in terms of the resistance, elasticity, and permeability, a TPP curing bath followed by a water bath were employed. Two kinds of membranes were obtained: Base membranes (without mechanical pores) and activated membranes (by creating mechanical pores as channels that communicate the three layers).

As previously reported by Uhoda et al., a pore is a term not clearly defined. It can have different meanings, depending on its aspect. Usually, invisible pores refer to the openings of the sweat gland apparatus. On the contrary, visible pores are enlarged empty funnel-shaped or cylindrical horny impacted openings, known as comedones, of pilosebaceous follicles [41]. Basically, they are apertures from the surface of the skin that ensure the input and output of gas and fluids [42]. Herein, pores are defined as channel connections between upper and lower layers.

**Figure 1:**
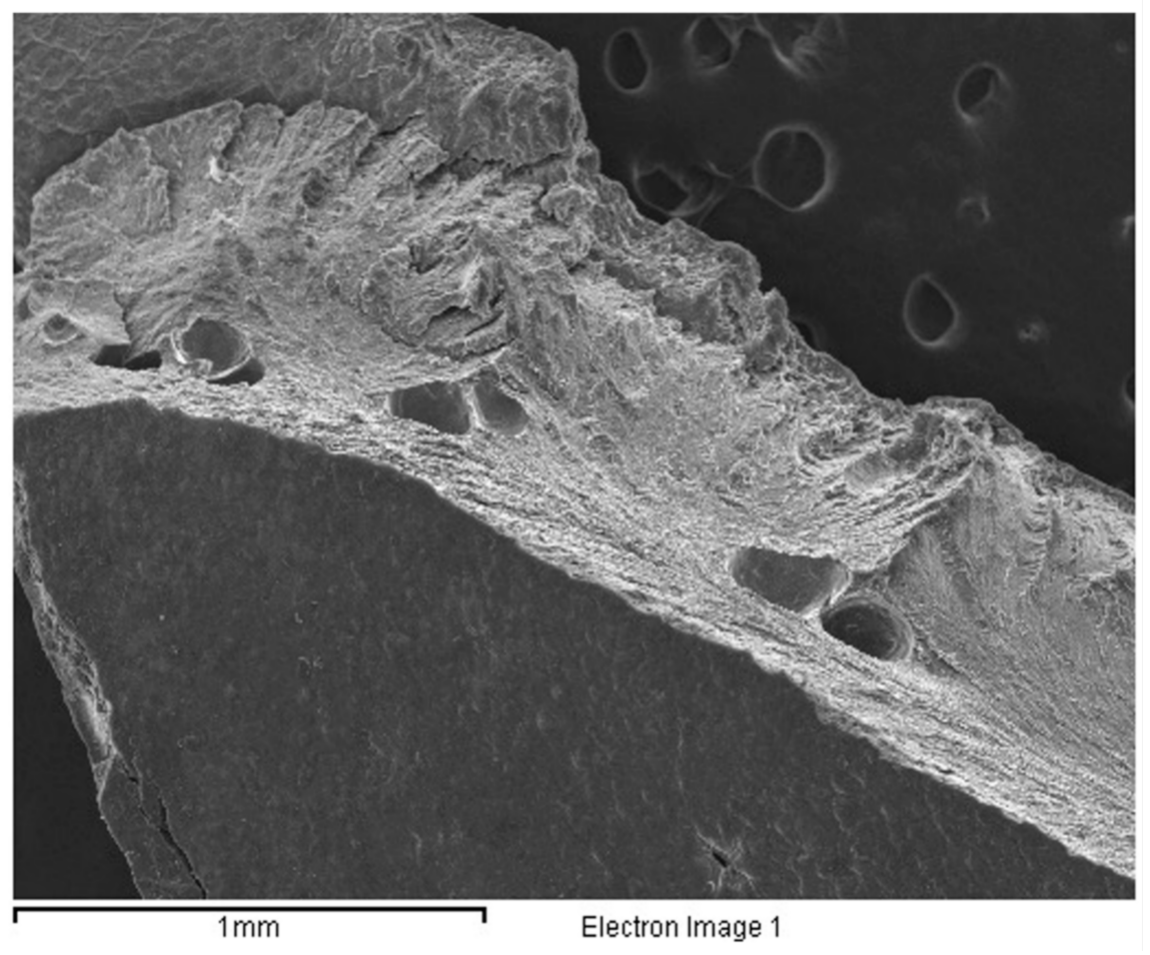
SEM image of a 3-layered crosslinked membrane profile with silica particles and subsequent dissolution.

In a first attempt to create an activated membrane, chemical methods of pore creation were studied by the addition of different porogens [43,44,45,46,47]. In our case, silica particles were used, but several difficulties were found when dissolving silica contained in the inner layer, despite the fact that different bath conditions were tested. As a consequence, channel formation was not feasible. Therefore, arbitrary pores were formed, as shown in Figure 1.

Considering this, a new method based on the creation of mechanical pores was used instead. A microneedling device, known as DermaStamp^®^, was employed to create the pores [48]. After placing the membranes in the TPP and water baths, the cured membrane was nailed with DermaStamp^®^. When checking pores with an optical microscope, heterogeneous pores with different shapes and areas were seen. Therefore, in a second attempt, a specific combination of TPP and water baths with DermaStamp^®^ was applied to the membrane, in order to achieve regular and homogeneous pores. By conducting the curation process at the same time as microneedles were pricking the membrane, for a specific period of time, the membrane scaffold tightened the structure to the surroundings of the microneedle. When checking pores with the optical microscope, roundish and same-size pores were achieved.

Therefore, pore creation succeeded with the 85% DDA chitosan membranes. In the case of 76% DDA chitosan membranes, as they retained a high quantity of water, pores were immediately filled with water once DermaStamp^®^ was removed. As a consequence, at the end, pores were irregular and were closed or almost closed. This has also been previously reported by Mengatto et al. [49]

Based on the results obtained in this study, the DDA and the different origins of chitosan influence the elastic modulus, and a low DDA has the highest impact in terms of reducing the elastic modulus of the membrane.

Additionally, the effect of the crosslinking between the different layers of the membrane was checked by testing its elasticity behavior. It has been stated that each layer brings a higher elasticity to the membrane when crosslinked in comparison with non-crosslinked layers.

The swelling behavior was also studied, being 2.5 times higher for the base membrane and 3.5 times higher for the activated membrane with the lowest DDA chitosan compared to the highest DDA chitosan studied.

## 2. Materials and Methods

### 2.1. Materials

Medium molecular weight chitosan of shrimp shell origin with a DDA of 76% and of seashell origin with a DDA of 85% were acquired from Aldrich, Merck Life Science S.L. (Madrid, Spain) (product number 448877) and medium molecular weight chitosan from snow crab with a DDA of 85% (Chitoscience chitosan 85/500, product number 23506) was obtained from Heppe Medical Chitosan GmbH (HMC+), (Halle, Germany). Chitosan of 76% DDA and chitosan of 85% DDA, both from Aldrich, were from Iceland and China, respectively. In the case of chitosan of 85% DDA from HMC+, it was from Canada. Acetic acid glacial and sodium hydroxide pellets were acquired from PanReac AppliChem, Panreac Química S.L.U. (Barcelona, Spain). Sodium tripolyphosphate with a technical grade of 95.2% was supplied by Alfa Aesar, Thermofisher GmbH (Kandel, Germany). Agarose BioReagent for molecular biology, low electroendosmosis (EEOO) was supplied by Sigma, Merck Life Science S.L. (Madrid, Spain). The phosphate-buffered saline tablet was supplied by Sigma, Merck Life Science S.L. (Madrid, Spain). One tablet of phosphate-buffered saline dissolved in 200 mL of deionized water yields 0.01 M phosphate buffer, 0.0027 M potassium chloride, and 0.137M sodium chloride, pH 7.4, at 25 °C. Calcium chloride from Quimivita, S.A. (Barcelona, Spain) was used in granular form. Teflon molds were fabricated at the university, and were especially designed for the creation of these membranes. The microneedle device was a 140 DRS DermaStamp^®^ system acquired through IBeautyMachine.com, GBS International holding Ltd. (Guangzhou, China). It was composed of 140 stainless steel microneedles; the needle width was 0.25 mm and the needle spacing was 1.58 mm. The depth of the needles could be adjusted from 0.2 to 3.0 mm, in order to assure that they crossed all membrane layers. In this work, a Dermastamp^®^ with needles of a 3.0 mm depth was employed. Therefore, complete perforation of the three layers of the membrane was achieved. Franz cells of a customized size were designed and fabricated by Fisher Scientific, S.L. part of Thermofisher Scientific (Madrid, Spain).

### 2.2. Methods

All experiments were conducted between 19 and 22 °C and 65 and 85% of relative humidity (RH).

#### 2.2.1. Support Preparation

Agarose was used as a support for membrane manipulation. It contains certain sulfates producing a slightly negative charge. For this reason, the agarose supports were soaked in a TPP bath, so that chitosan, with a positive charge, interacted with the residual TPP, with a negative charge, and not with the supports directly.

Agarose 2% (*w*/*w*) solution was prepared to obtain flexible supports used in membrane preparation, magnetically stirred, and heated up to 80 °C until the solution turned from white to totally transparent.

Afterwards, 9 g of the hot solution was placed in Teflon molds for 5 min to obtain membranes with the same form and diameter. Then, agarose supports were immersed in a bath of 10% TPP (*w*/*w*) for at least 15 min to passivate agarose reactivity and a water bath for 15 min to remove excess TPP. By conducting this procedure, chitosan solution can be prevented from getting fixed to the agarose support. Therefore, it can help with agarose support release once the layer is formed.

The resulting gelatinized agarose supports could be reutilized for 3–4 weeks until their degradation. For each membrane, at least two agarose supports were required.

#### 2.2.2. Solution Preparation

For the purpose of obtaining base and activated membranes, three solutions were needed.

Solution 1 is an acetic acid 2% (*v*/*v*) solution and Solution 2 corresponds to 1 g of NaOH in 25 mL of water. Both solutions were used to prepare the chitosan solution.

Solution 3 was a 10% sodium tripolyphosphate (TPP) (*w*/*w*) solution and was prepared with magnetic stirring until TPP dissolution. This solution was reacted with chitosan and employed to produce a crosslinking reaction in the membrane preparation procedure.

A total of 3 g of chitosan powder was added to 97 mL of acetic acid solution. Then, different quantities of NaOH solution were added, depending on the DDA of chitosan. The total solution was firstly stirred manually, for about one minute, until jellification was obtained. Subsequently, it was stirred automatically with an anaxial flow agitator for 3 h at 542 rpm until homogenization.

The pH of the solution was measured in order to verify that was between 5.5 and 5.8 for chitosan 85% DDA and 4.8–5.0 for chitosan 76% DDA. This difference in pH margins is due to the different DDA of chitosan. These pH margins assured that the crosslinking reaction between layers took place. A weight correction of chitosan solution was made by adding water, in order to always obtain a 116 g total dissolution weight, and it was stirred with an anaxial flow agitator for 15 min at 542 rpm until homogenization. Then, it was ready to use.

#### 2.2.3. Membrane Preparation

##### Tri-Layered Membrane Preparation

Preparation of the base membrane consisted of three steps, in order to create three layers. First, to simulate the deepest layer, 4 g of chitosan solution was weighted in an agarose support and was homogenously distributed.

The other agarose support was positioned in a Teflon mold and 2.15 g of 10% TPP (w/w) was impregned with a syringe or dropper covering the whole agarose surface. Chitosan solution distributed in the agarose support was placed on top of the agarose impregned with TPP for 5 min, in order to guarantee the crosslinking. A Teflon lid of 265 g was put on top so as to improve the membrane flatness. After this period of time, the lid and upper agarose support were withdrawn to obtain the first membrane layer.

The second layer was prepared by adding 2.15 g of TPP solution to the first layer, as was done for the first layer, in order to crosslink both. Then, 3 g of chitosan solution was added again in an agarose support and placed on top of the first membrane layer for 5 min. A Teflon lid was again placed on top. The lid and upper agarose support were then withdrawn, producing the second membrane layer, and another 2.15 g of TPP solution was added to produce the last crosslinking.

The last layer was prepared by adding 3 g of chitosan solution in an agarose support, which was placed on the upper surface of agarose already impregned with TPP for 5 min, with the Teflon lid on top.

Finally, the upper agarose support was removed and 2.15 g of TPP solution was added in the last layer. To obtain consistency, the membrane was maintained for 4 h in the Teflon mold with the upper Teflon lid. A bubble leveler was needed for controlling the flatness and correcting the level, if necessary, at the beginning of the 4 h. Once the time was up, the membrane was weighted and kept in a Petri capsule. The next step was performing a curation process, which is explained in this section.

**Scheme 1:**
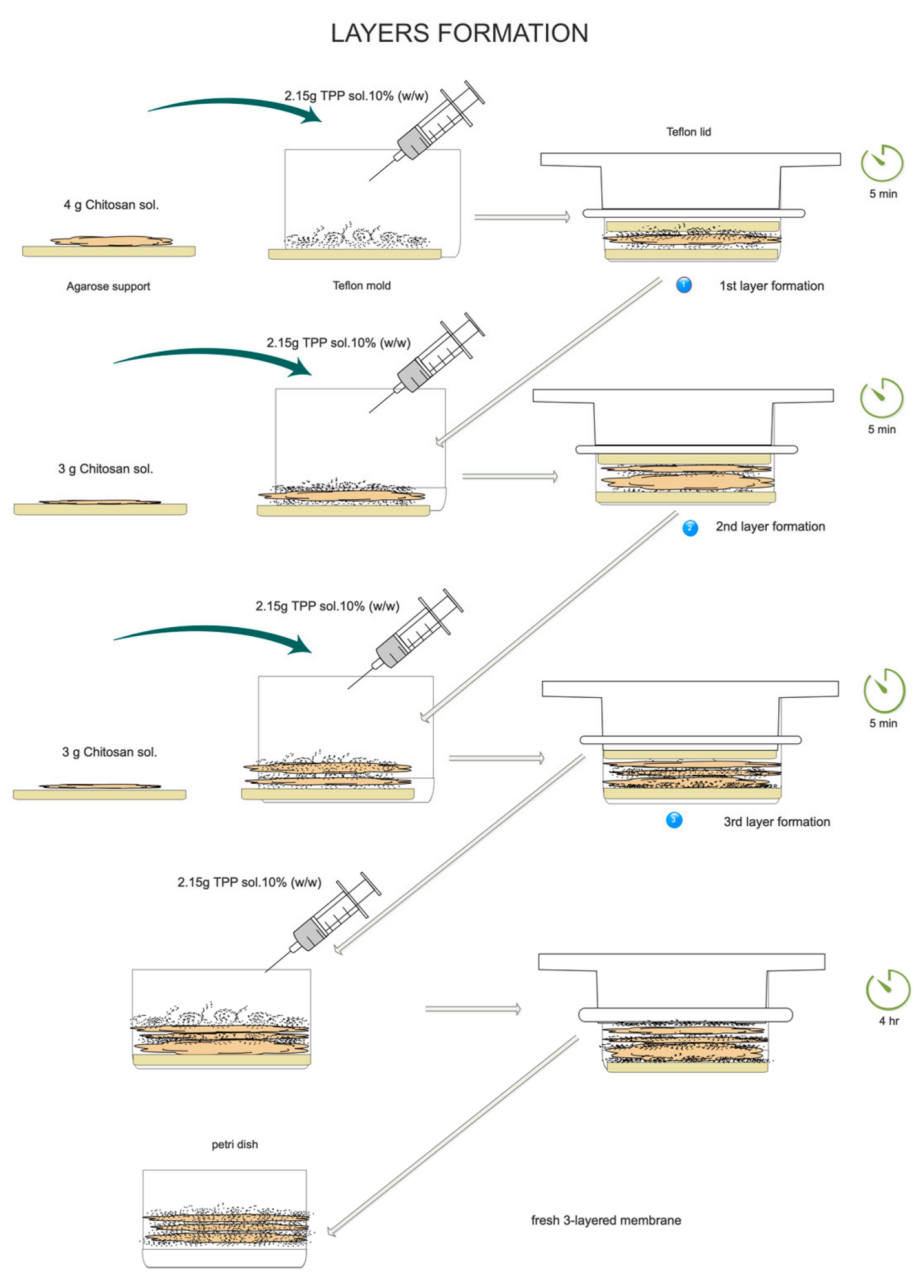
Tri-layered crosslinked membrane formation.

The above description is summarized in Scheme 1.

##### Membrane Curation Process

The curing process was different for base and activated membranes.

##### Base Membrane Curation Process

**Scheme 2:**
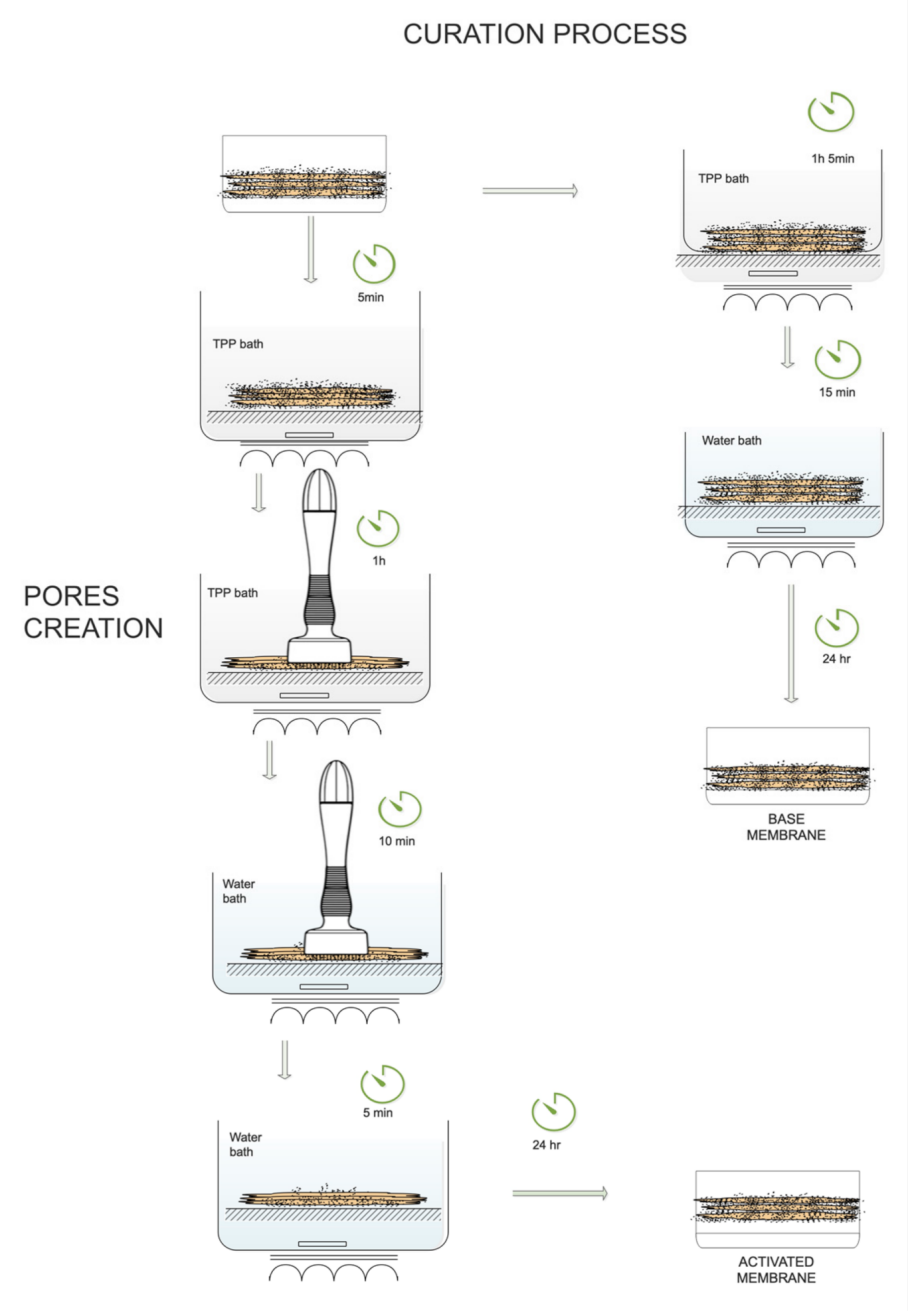
Curation process of activated and base tri-layered membranes.

The last step in obtaining a crosslinked membrane and improving its resistance was to soak it in a 10% (*w*/*w*) TPP solution bath for 1 h and 5 min, followed by a 15 min water bath, with both baths exhibiting magnetic stirring, in order to neutralize the pH. Then, the membrane was placed in a Petri dish and left to rest for 24 h. The curation process is summarized in Scheme 2.

##### Activated Membrane Curation Process

Once the membrane was formed, it was soaked in a 10% (*w*/*w*) TPP solution bath for 5 min in order to improve the membrane consistency. Following this, it was removed from the bath and punched with a microneedling device known as DermaStamp^®^. The depth of the microneedles could be regulated up to 3.0 mm. In this work, a 3.0 mm length was used, so a complete perforation of the membrane was achieved. An agarose support located below the membrane was required during the process, in order to prevent microneedle breakage. When punching, it was necessary to make sure that all needles had crossed the membrane by checking it through the transparent agarose. If necessary, we checked that all the microneedles crossed the membrane by applying a small amount of pressure through the agarose. Then, the membrane was soaked again in the same TPP bath for 1 h with DermaStamp^®^ nailed. Afterwards, a 10 min water bath was employed with DermaStamp^®^ still nailed, in order to stop the TPP reaction. Following this, the microneedles were removed by rolling the adjuster up to the 0 position, in order to avoid membrane tearing. Finally, a 5 min water bath without DermaStamp^®^ was performed, in order to neutralize the whole membrane. The above description is summarized in Scheme 2.

After 24 h, the membrane was cut with a sharpened metal cylinder of a 2 cm diameter in order to get rid of irregular outlines. Its thickness was measured at four different sides with an electronic digital caliper so as to assure a quite homogeneous level across the whole membrane. Then, it was weighed again before the experiment.

##### Bi-Layered Membrane Preparation

The preparation of the bi-layered membrane was the same as the one explained for the tri-layered membrane but, in this case, once the second layer was impregned with the 2.15 g of TPP solution, the Teflon lid was put on top. As has already been explained, it was left for 4 h and followed by the curation process of the base or activated membrane.

##### Mono-Layered Membrane Preparation

The preparation of the mono-layered membrane was the same as the one explained for the tri-layered membrane but, once the first layer composed of 4 g of chitosan solution was impregned with the 2.15 g of TPP solution, the last step consisted of putting the Teflon lid on top. As already explained, it was left for 4 h and followed by the curation process of the base or activated membrane.

### 2.3. Characterization

The different kinds of membranes obtained have a special identification system. An example for membrane identification (ID) is presented below:

M3B-C85A-TX

First position: M = membrane

Second position: 3, 2, 1 = number of layers

Third position: B = base membrane or A = activated membrane

Fourth position: C = chitosan

Fifth position: 85 or 76 = %DDA

Sixth position: A or H = Aldrich or HMC + supplier

Seventh position: T from TPP crosslinker

Eighth position: X or 0 = crosslinked or non-crosslinked

All characterization experiments presented below were carried out with tri-layered crosslinked membranes, except for some rheology tests, which were expressly mentioned.

#### 2.3.1. Pore Quantification

**Figure 2:**
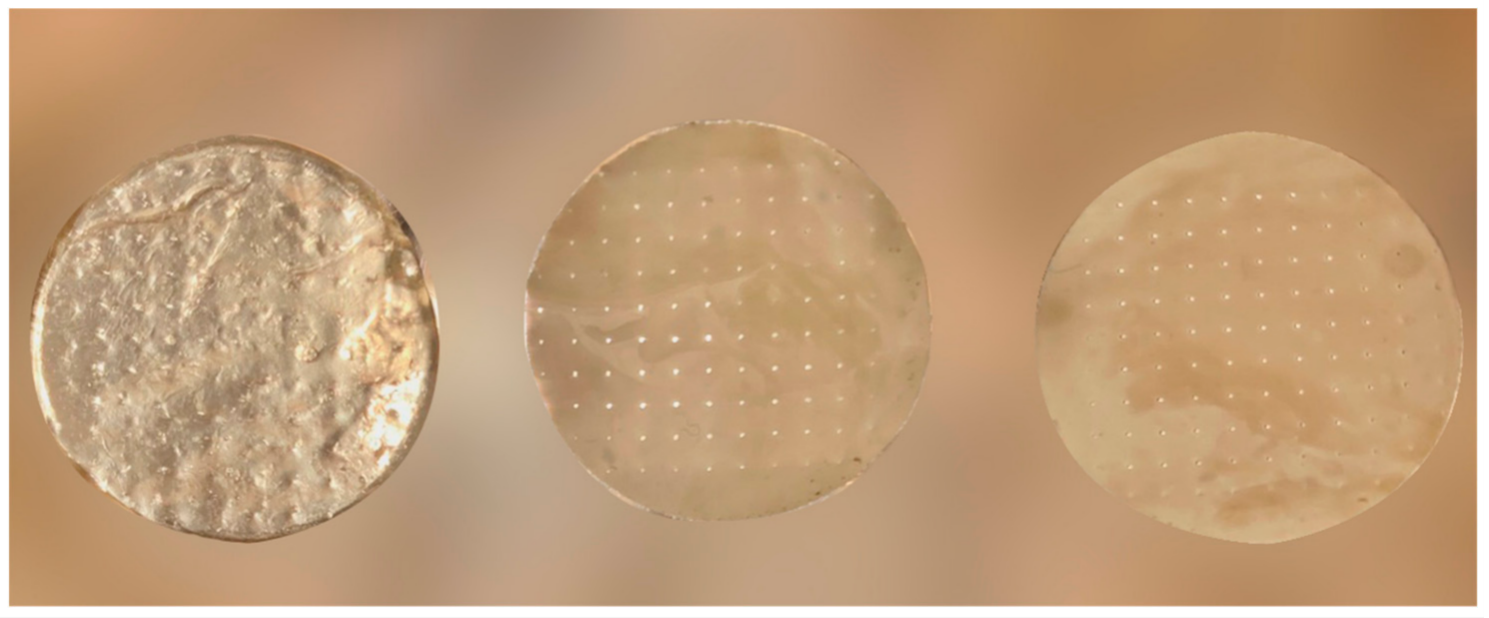
Appearance of tri-layered crosslinked membranes after 24 h. ID from left to right: M3A-C76A-TX; M3A-C85H-TX; and M3A-C85A-TX.

The quantification of pores was conducted on the following membranes: M3A-Q85A-TX and M3A-Q85H-TX as pore channels were more evident in those kinds of membranes. This can be observed in Figure 2. No pores were properly formed for M3A-Q76A-TX due to the highest water capture of the membrane following the curation baths, which closed most of the pores. The microneedle marks were observed, but only some randomly small pores were formed.

Pores of the tri-layered crosslinked membranes were observed through a Euromex Bioblue Optical Microscope. A semi plan 4x/0.10 oil immersion objective of a wide field of 0.45 was used.

For measuring the area of pores, the ImageFocus 4 software was required. The calibration correction was applied on the computer with the objective used. The resolution chosen was 2040 × 1528. The pores observed were located in the diffusion area of Franz cell permeation tests. The number of pores located in the 1.06 cm2 diffusion area was 52.

**Figure 3:**
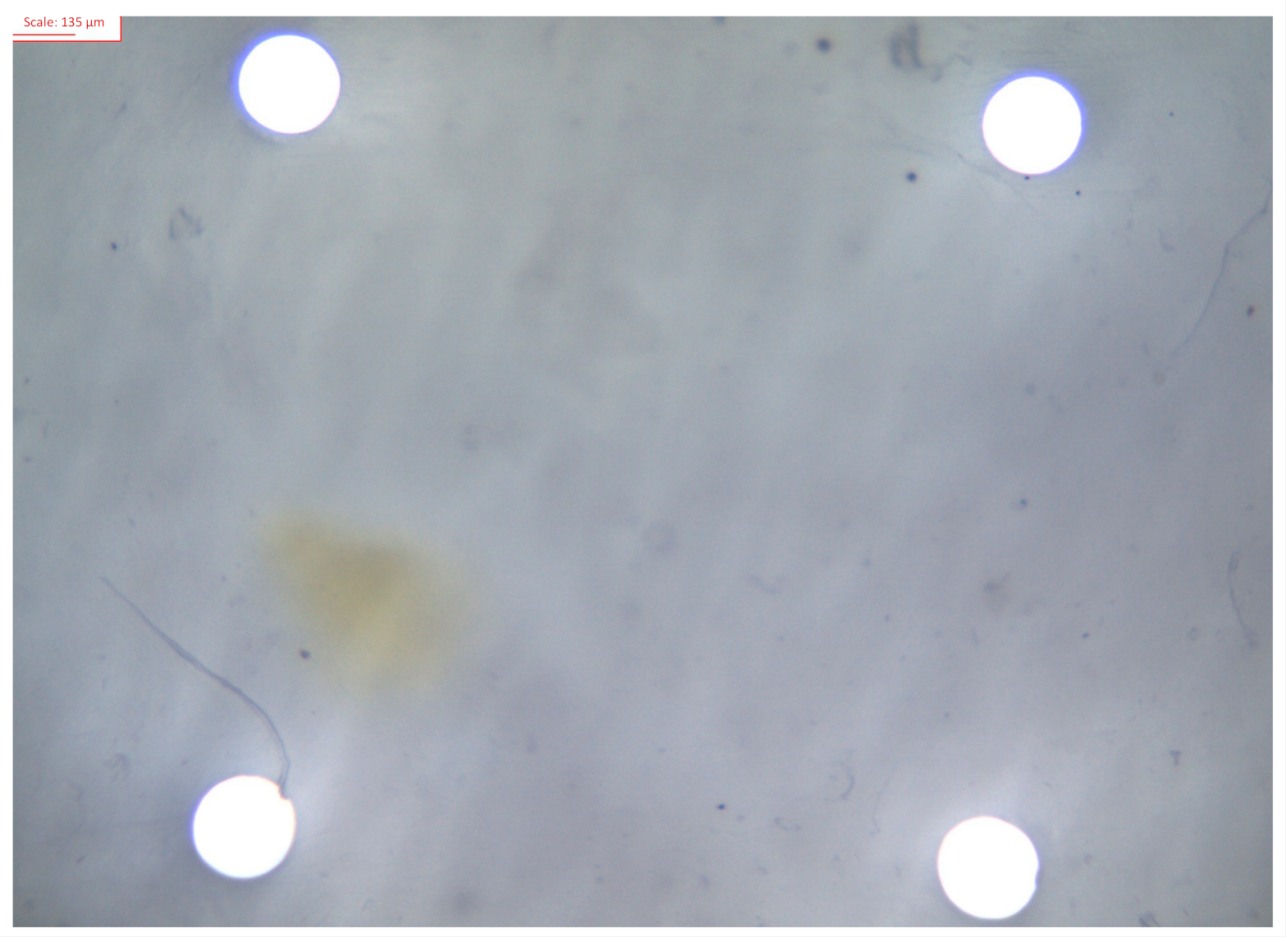
Mechanical pores observed with the Optical Microscope for M3A-C85A-TX.

As can be visualized in Figure 3, periodical pores were seen throughout the activated tri-layered crosslinked membranes of 85% DDA.

#### 2.3.2. Permeation Tests

The water permeation tests were performed with customized Franz cells, from Fisher Scientific, S.L. (Madrid, Spain), especially designed for the membranes under study. The donor compartment had a 30 mL capacity. The diffusion area was 1.06 cm^2^.

**Figure 4:**
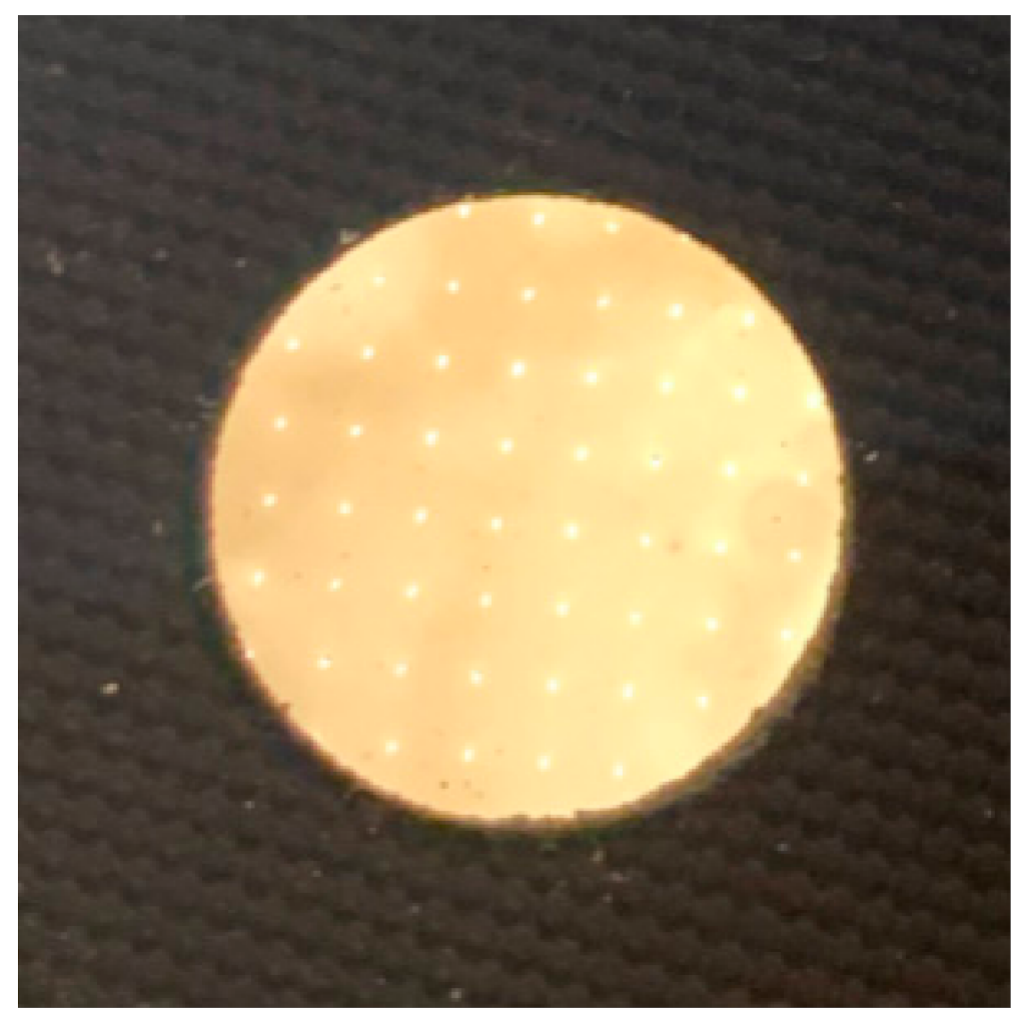
Activated tri-layered crosslinked membrane of 85% degree of deacetylation (DDA) chitosan located within two diffusion rings.

The membrane needed to be fixed between two permeation rings and collocate the rings between donor and receptor compartments from Franz cells, as can be observed in Figure 4. Then, the system was sealed and clamped until estanqueity. Therefore, 30 mL was introduced in the donor compartment.

#### 2.3.3. Scanning Electron Microscopy (SEM)

The membranes obtained were dried with air exposure until reaching a constant weight at room temperature (19–22 °C and 65-85% of RH).

The SEM was a Jeol JSM 5310, from Jeol Ltd. (Tokyo, Japan) with Oxford Inca Energy software, version 4.09 (Oxford, UK). Sample preparation was performed by producing a metallic coating with a sputtering Polaron SC 7620 Sputter Coater from Quorum Technologies Ltd, (Laughton UK), with 8-10 mbar of Ar pressure and a target of Au. In these conditions, the coating had a thickness between 150 and 200 Angstroms.

#### 2.3.4. Rheology Tests

Rheology was carried out for base and activated membranes with different layers combined with a 20 mm diameter, with an AR 2000 ex Rheometer and Rheology Advantage Instrument Control AR, both from TA Instruments, (New Castle, DE, USA). The conditions were a 1 Hz frequency, strain between 0.01 and 100%, a 20 mm steel cross-hatched (CH) plate, and 25 °C. In the test, the membrane was put on top of the lower geometry and the rheometer motor decreased the upper geometry until the normal force applied was around 1.5 N. The elastic modulus of the material was obtained with an oscillatory procedure.

#### 2.3.5. Swelling Index

After 24 h of membrane creation, the samples were dried for 24 h in a container which contained granular calcium chloride. By this time, it had already been checked that the membrane weight was constant, so it was assured that the samples were completely dried.

**Figure 5:**
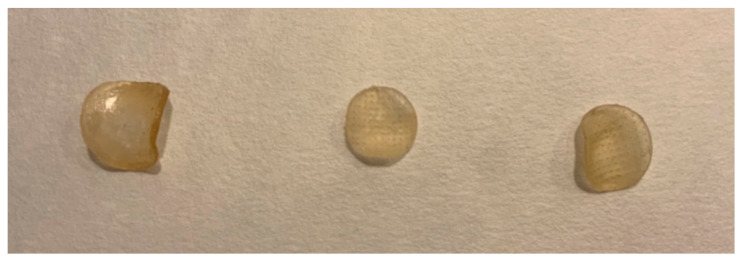
Dehydrated tri-layered activated crosslinked membranes, from left to right: M3A-C76A-TX; M3A-C85H-TX; and M3A-C85A-TX.

In Figure 5, the dry aspect of the tri-layered activated crosslinked membranes can be observed.

The dried membranes were weighed and immersed in phosphate buffered solution (PBS, pH 7.4) at different times until reaching the maximum weight. Phosphate-buffered solution was prepared as previously reported. The membranes were immersed in phosphate buffered solution under constant agitation during the entire experiment.

**Figure 6:**
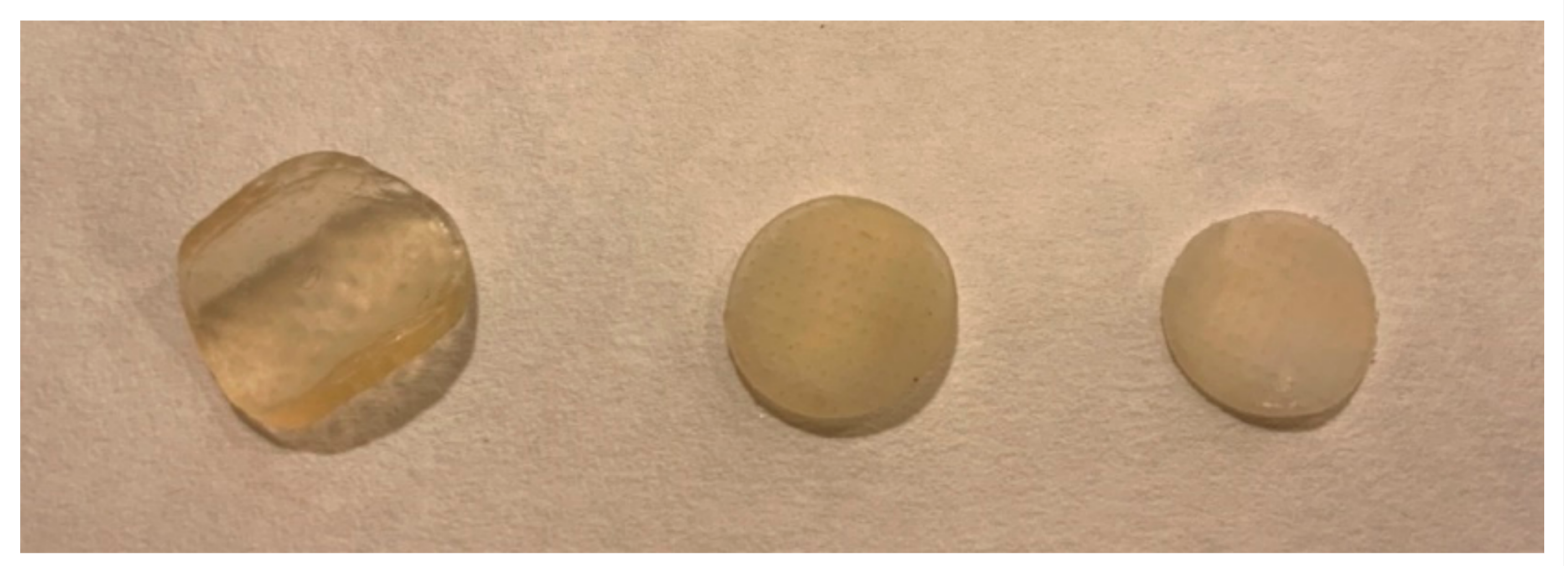
Swollen tri-layered activated crosslinked membranes, from left to right: M3A-C76-TX; M3A-C85H-TX; and M3A-C85A-TX.

The maximum swollen aspect of the different kinds of tri-layered activated crosslinked membranes can be observed in Figure 6.

The weight of the swollen sample was measured with an electronic balance after the surface water was removed with filter paper. Then, the swelling index (SI) of the membranes was calculated by Equation (1):

(1) $$\mathrm{SI}\;\left(\%\right)=\frac{\mathrm{Ws}-\mathrm{Wd}}{\mathrm{Wd}}\times 100$$

where Wd and Ws are the weights of dried and swollen samples, respectively.

## 3. Results and Discussion

Once tri-layered crosslinked membranes were cut with the 2 cm diameter cylinder, their weight and thickness were recorded (Table 1).

As can be seen, activated membranes were a bit thinner and had a slightly lower weight than base membranes. This was due to the application of DermaStamp^®^ during the membrane curation process. Membranes of 76% DDA had a higher weight than 85% DDA membranes as they tended to trap more water during the curation baths.

### 3.1. Pore Quantification

The area of pores measured is shown in Table 2 and is calculated as the area of a circle.

As can be observed, the mean pore area can be considered the same when comparing membranes created with the two different kinds of chitosan of 85% DDA.

The size of pores obtained was compared with the results of different human skin previously reported in scientific journals [42]. As can be viewed in the referenced paper, depending on the human race, the facial pore size can vary from 15,000 µm^2^ for the Chinese race up to 92,500 µm^2^ for the Brazilian race. As has already been shown in other studies, it can be highlighted that Asiatic facial pores represent the race with the smallest pores and Caucasian skin values are in the middle, being between 40,000 and 55,000 µm^2^, calculated as the area of a circle [50].

### 3.2. Permeation Tests

A permeability test was conducted for both base and activated tri-layered crosslinked membranes for the different chitosan, using a Franz diffusion cell 24 h after membrane creation. In order to study the permeability, 30 mL of water was added in the Franz diffusion cell.

For both types of membrane, the water permeated in the receptor compartment was collected by the sampling port with a syringe at different times and placed in a beaker previously tared. The amount of water which permeated was plotted over time, as is shown in Table 3 and Table 4.

**Figure 7:**
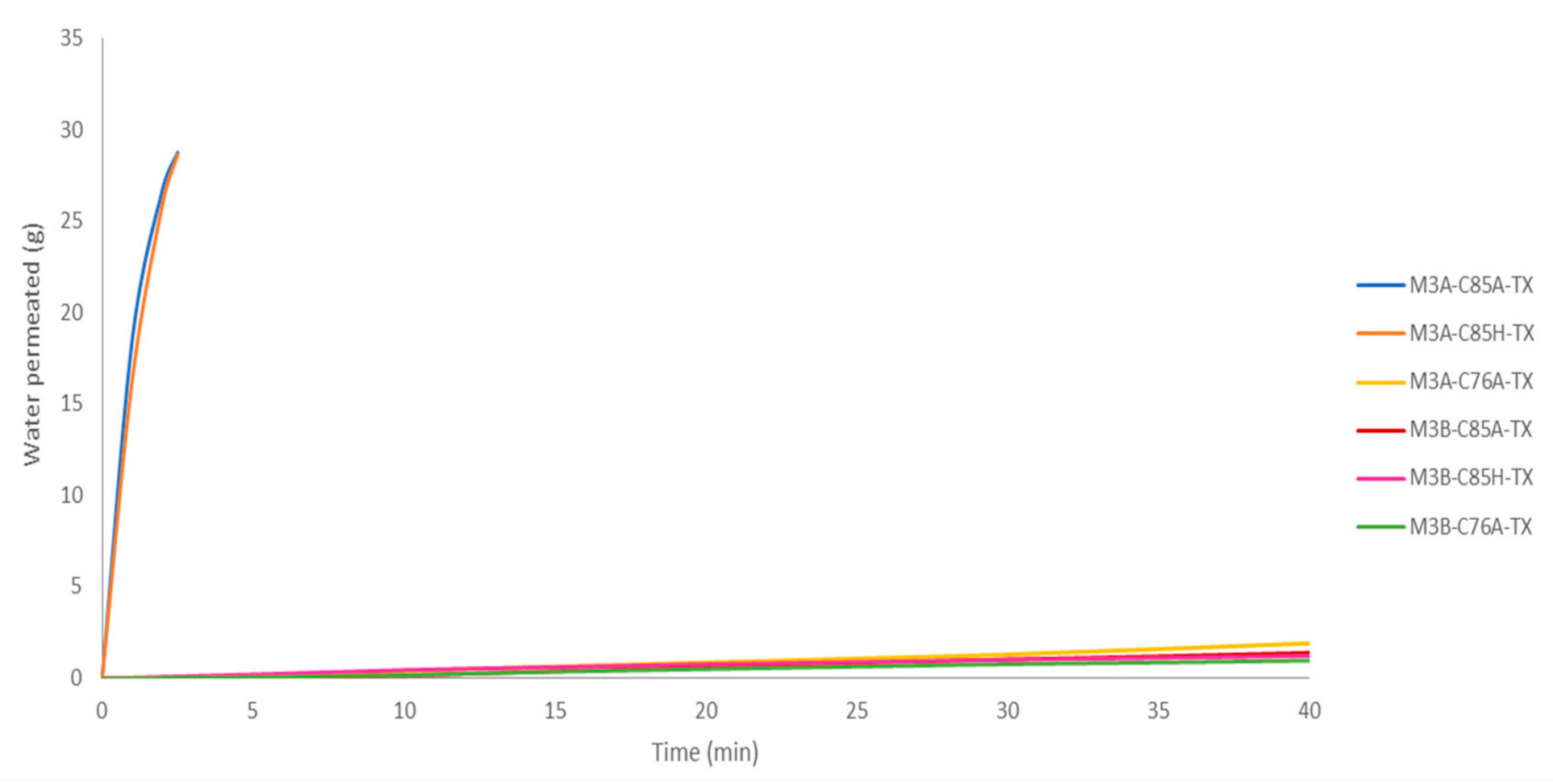
Mean water permeation through activated and base tri-layered crosslinked membranes for the three kinds of chitosan studied.

Two clearly differentiated tendencies were observed from activated and base tri-layered crosslinked membranes for chitosan 85% DDA. On the contrary, almost no differences were found between activated and base membranes for chitosan 76% DDA activated tri-layered crosslinked membranes (Figure 7). This is in line with what other authors have previously reported [49].

### 3.3. Effect of DDA and the Origin of Chitosan on the Properties and Aspects of the Membranes

Membranes created with 85% DDA chitosan are more elastic and more flexible, and have more orifices in their structure. Flexible chains of chitosan favor hydrogen bonding formation as more glucosamine groups facilitate their formation. On the other hand, 76% DDA chitosan has more acetyl groups that block the hydrogen-bonding formation.

Hydroxyl and amine groups from chitosan cause the swelling of chitosan membranes. A higher DDA of chitosan will lead to a higher amine group content, and thus to a higher level of swelling. However, as already reported by Wan et al. [51], it has been proven that swelling decreases when DDA increases. The explanation for this is related to the kind of hydrogen bond formation. When applying a greater DDA, the intramolecular hydrogen bonds between hydroxyl and amine groups are much stronger than for intermolecular groups between them and water molecules. As a consequence, the high crystallinity of the 85% DDA chitosan membrane prevents water from entering those sections and blocks the swelling of the membrane.

Therefore, with an increase of the DDA, it has been shown that the swelling index and elastic modulus decrease. These aspects agree with what has already been reported by Cao et al., who also proved that the tensile strength increases [29].

#### 3.3.1. Scanning Electron Microscopy

**Figure 8:**
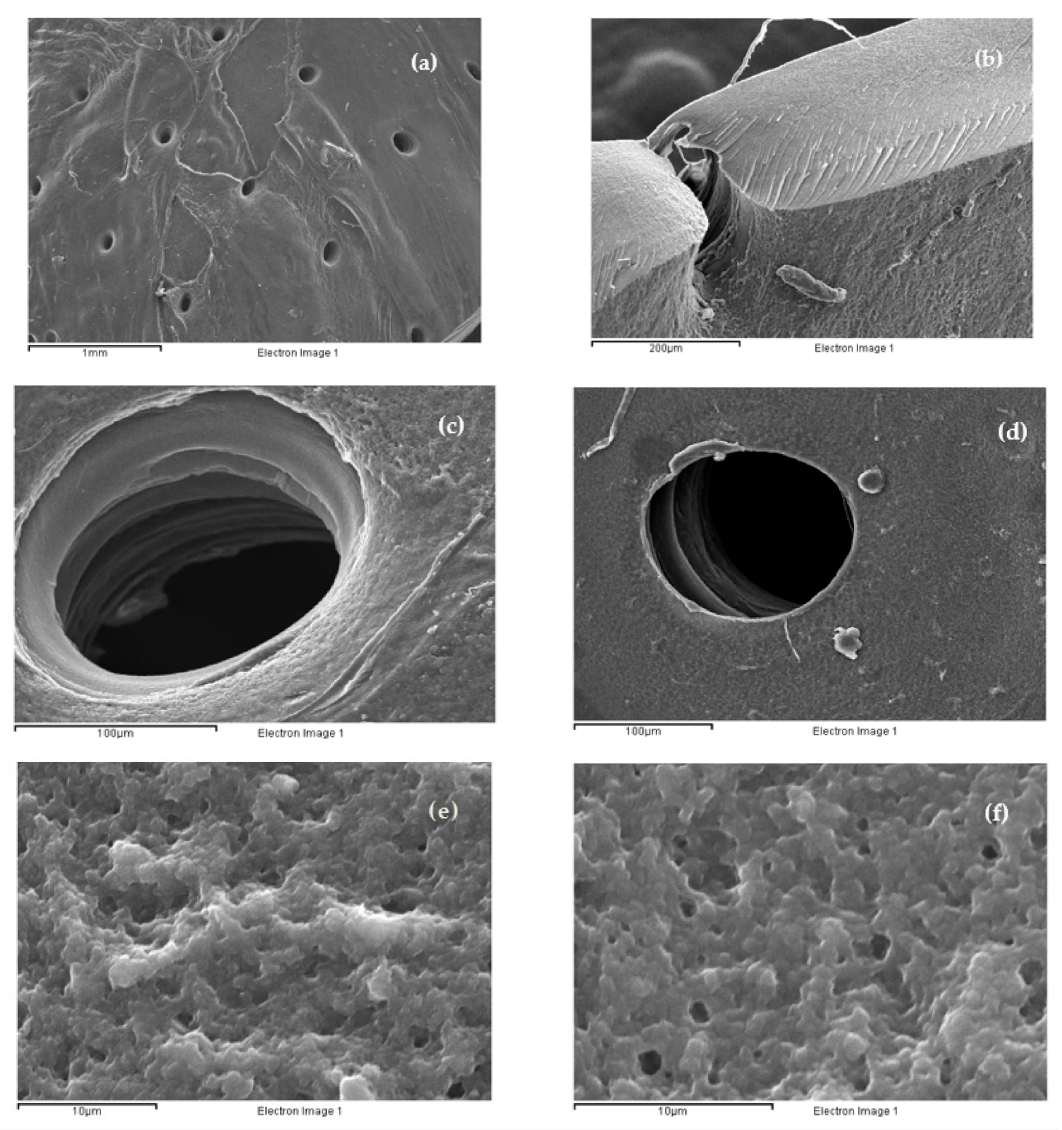

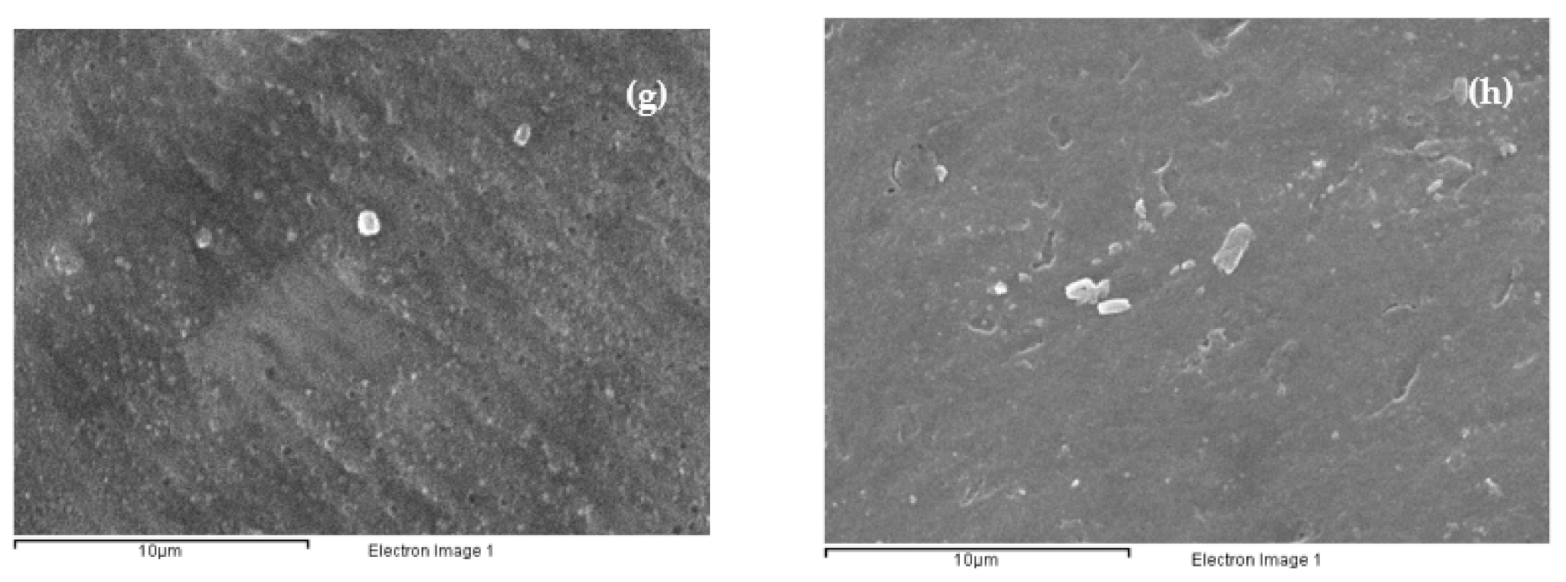
SEM images of the tri-layered crosslinked membranes with the different chitosan: (**a**) Top M3A-C85A-TX; (**b**) profile M3A-C85H-TX; (**c**) top M3A-C85H-TX; (**d**) bottom M3A-C85H-TX; (**e**) top M3B-C85H-TX; (**f**) bottom M3B-C85H-TX; (**g**) top M3B-C76A-TX; and (**h**) bottom M3B-C76A-TX.

Figure 8 presents the morphology of the different activated and base tri-layered crosslinked membranes with different chitosan.

The SEM image of Figure 8a for activated tri-layered crosslinked membranes with 85% chitosan shows the shape of the mechanical pores marked periodically in the membrane. In Figure 8b, the profile of the membrane is shown and slightly different textures can be observed. Additionally, a cross-sectional pore can also be observed. One pore can be observed in depth in Figure 8c,d, from the top and bottom, respectively.

In Figure 8e,f, where membranes are created with chitosan 85% DDA, it can be seen that a fluffier surface was obtained. These surfaces correspond to the top and bottom sides of the membrane, respectively, and several orifices can be appreciated. On the other hand, a dense and flat surface is observed in Figure 8g,h, belonging to chitosan of 76% DDA.

#### 3.3.2. Elastic Properties of the Different Tri-Layered Crosslinked Membranes

The rheometer represents an advantage for testing the elasticity in ex-vivo human skin as this can be tested under different stress values and rates. Additionally, human skin can be tested until failure and more information about it can be obtained. The stress strain relationships can also be modeled and quantified. However, ex-vivo human skin presents some drawbacks, as has previously been mentioned, such as sample preservation, ethical concerns, and difficultness in handling and holding the skin due to the softness of the tested tissue [52]. Therefore, artificial scaffolds, such as these tri-layered crosslinked membranes, are presented here as an alternative for testing different elasticity modulus values.

This section presents the results of base and activated membranes with three different types of chitosan: chitosan 76% Aldrich (C76A); chitosan 85% Aldrich (C85A); and chitosan 85% HMC+ (C85H). The mean of the obtained results is shown in Table 5 until 2 min, at which point the elastic modulus is nearly constant. In this way, it is possible to obtain information on the elasticity produced by each chitosan in the membranes and the variations of the elastic modulus with respect to activated and base membranes.

Standard deviation observed in Table 5 indicates that the procedure developed in this research is reproducible for every type of membrane.

**Figure 9:**
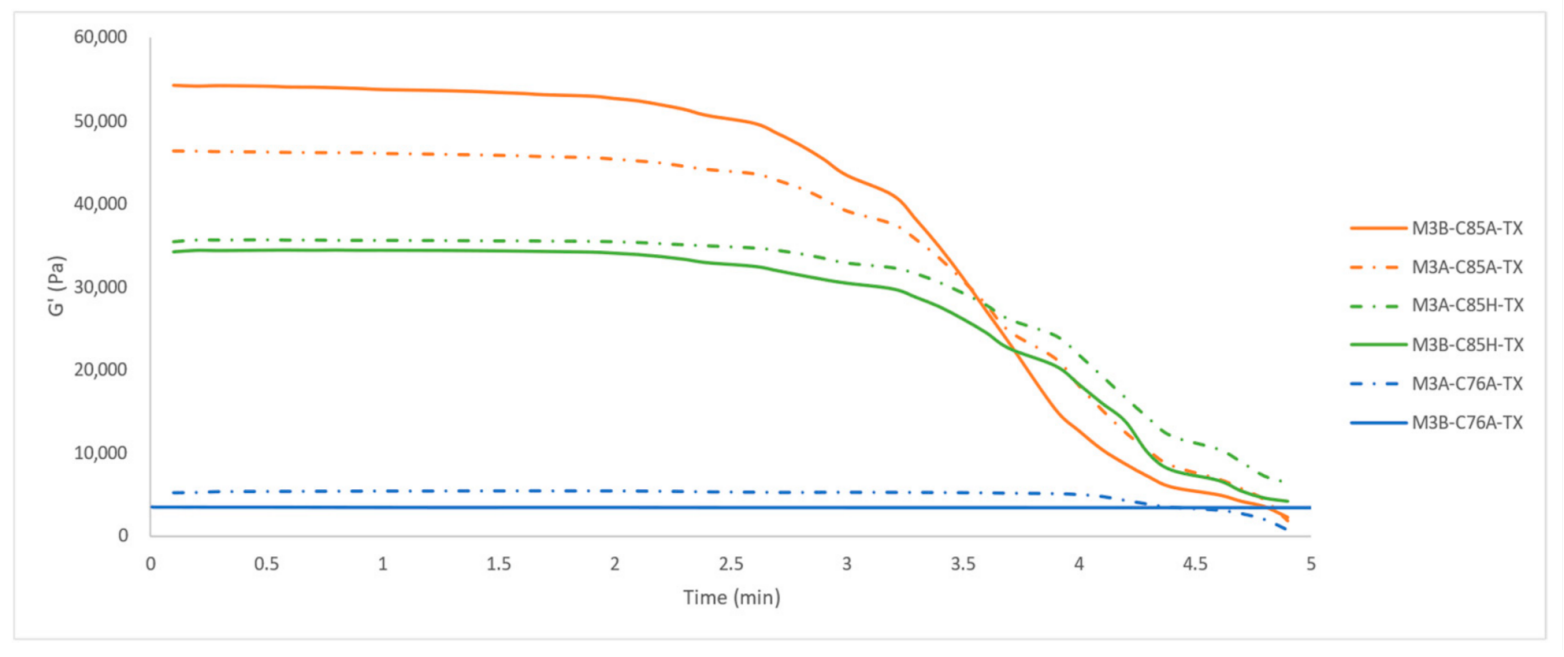
Mean elastic modulus for base and activated tri-layered crosslinked membrane with the three different kinds of chitosan.

Figure 9 shows that, for C85A, the highest elastic value was obtained. Specifically, base membranes had a higher elastic modulus than activated membranes. Instead, for C76A and C85H, there were no significant differences in elasticity between both activated and base membranes. These results establish substantial differences in the elastic modulus between the membranes created with the three different types of chitosan, and demonstrate that C85A has the highest impact in terms of elasticity.

For C85A, there was a significant difference in the elastic modulus between base and activated membranes. These results lead us to think that, since microneedles nailed the most elastic membranes, the pores influenced the membrane structure, breaking the elastic bonds. Therefore, the membranes lost their original shape and did not return to it, leading to a lower elasticity. On the other hand, for C85H and C76A, there is no significant difference between activated and base membranes. In these cases, the elasticity was not high enough to notice a remarkable difference when breaking the original structure of the membrane.

In this study, it has been observed that the different origin of chitosan can affect the elastic modulus of the membrane with the chitosan 86% Aldrich (seashell origin) being the most elastic, followed by chitosan 85% HMC+ (crab origin). The different elasticity that just a natural origin of chitosan can bring to the membrane was previously stated by Shepherd, R. et al. [27]

The chitosan of 76% had the lowest elasticity. This is due to the low DDA and the chitosan origin.

Much divergence has been found between different authors when comparing the elastic modulus of different human skin and different techniques, such as tensile tests, indentation tests, and suction or torsion tests [52,53,54]. As is mentioned in previous studies, a wide range of elastic modulus values, from 1000 to 57,000,000 Pa, have been reported. This large margin of the elastic modulus reaffirms the need for a human skin alternative with reproducible results.

A margin of elastic modulus from 3500 to 55,000 Pa was achieved with our tri-layered membranes, depending on the chitosan used, exhibiting a good reproducibility with each chitosan used.

A different behavior was found in our results and results obtained by Dunn and Silver [55] and Tongue et al. [56] In their study, the elastic modulus in lower strains was much lower than the elastic modulus in higher strains [52]. With the membranes used in this research, the behavior was the opposite, being higher in lower strains than in higher strains.

Some elastic moduli of the same order measured in vivo were found in previous studies. Bader and Bowker reported different values of the order of 1000 Pa for young and aged forearms. Zhang et al. reported values from 6630 ± 3.4 Pa for the back site up to 28,410 ± 13.32 Pa for the palm site. Other studies, as reported by Sanders et al., have stated a margin of 30,000-100,000 Pa for the forearm (<30 years) and 20,000–50,000 Pa for the forearm (>30 years) [57].

All these margins could be covered with the different chitosan studied here. Chitosan of 76% DDA would suit Bader and Bowker’s study and both kinds of chitosan of 85% DDA would cover the margin established by Sanders.

The effect of hydration on elasticity has also been previously studied. At low hydration levels, it was observed that the elasticity increased due to the stretching of bonds, but, at higher hydration levels, the hydrogen bonds became hydrated, and as a consequence, weakened [53]. This effect was also observed with chitosan of 76% of DDA. As it was quite extensively hydrated, the elasticity values were reduced by an order of 10 compared to C85A.

#### 3.3.3. Effect of the Crosslinking Grade of Chitosan with TPP on the Properties of the Membranes

The following combinations of layers were checked with the rheometer for both base and activated membranes for the two DDA of 76 and 85% from Aldrich (the latter represented 85% DDA): one layer, two layers, and three layers, both DDA non-crosslinked and cross-linked. In this way, it was possible to obtain information from the elasticity values about the crosslinking effect of the different layers and the effect of adding more layers to the membrane.

For chitosan 76% Aldrich (C76A), the mean of the obtained results for activated and base membranes is represented in Table 6 and Table 7, respectively, until 2 min (when the elastic modulus is nearly constant).

**Figure 10:**
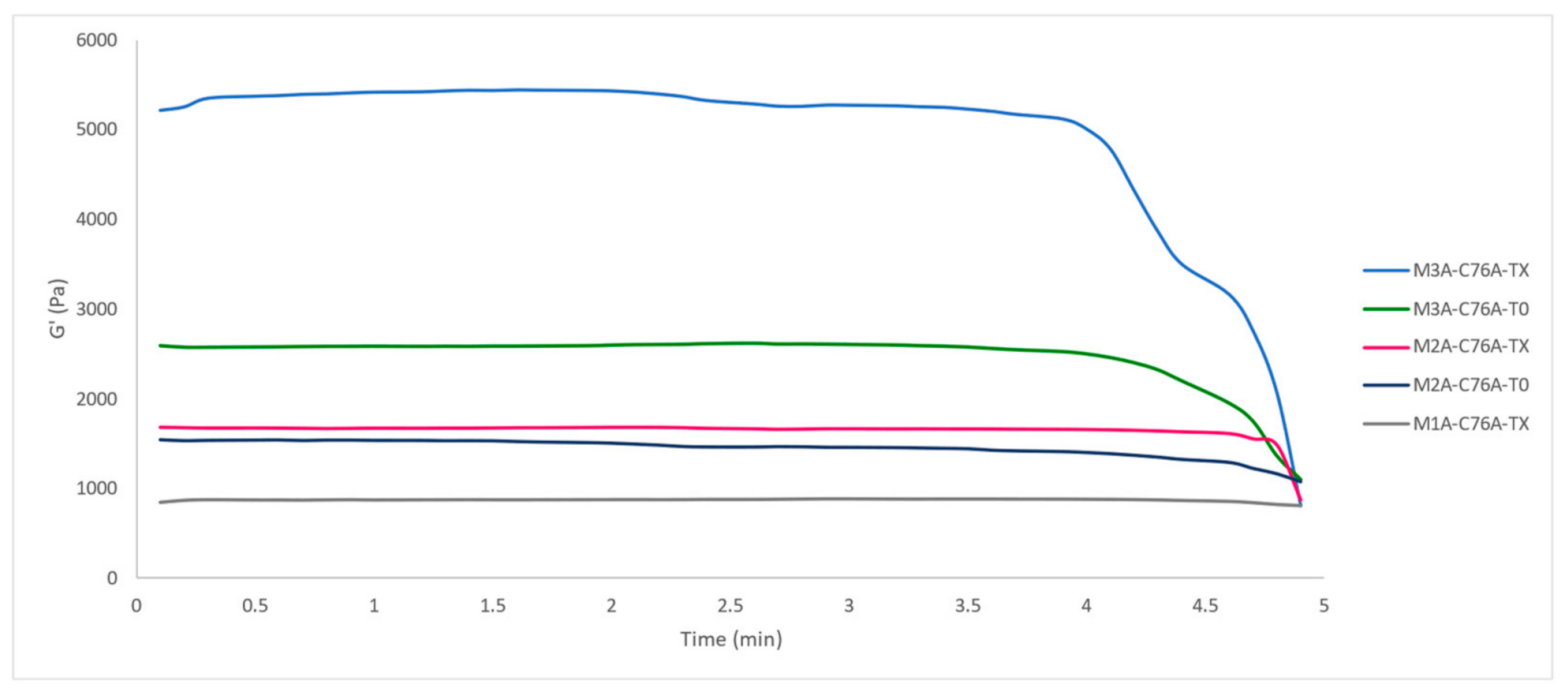
Mean elastic modulus for combinations of layers of activated membranes with 76% DDA chitosan from Aldrich crosslinked and non-crosslinked.

As can be seen in Figure 10, the most elastic membrane is the activated tri-layered membrane with crosslinking (M3A-C76A-TX). The following membrane is the activated tri-layered membrane without crosslinking. Following this, at around 1600 Pa, activated bi-layered membranes with and without crosslinking were found, with no significant difference. To end with, as was expected, there were membranes with less elasticity, which were the mono-layered membranes.

**Figure 11:**
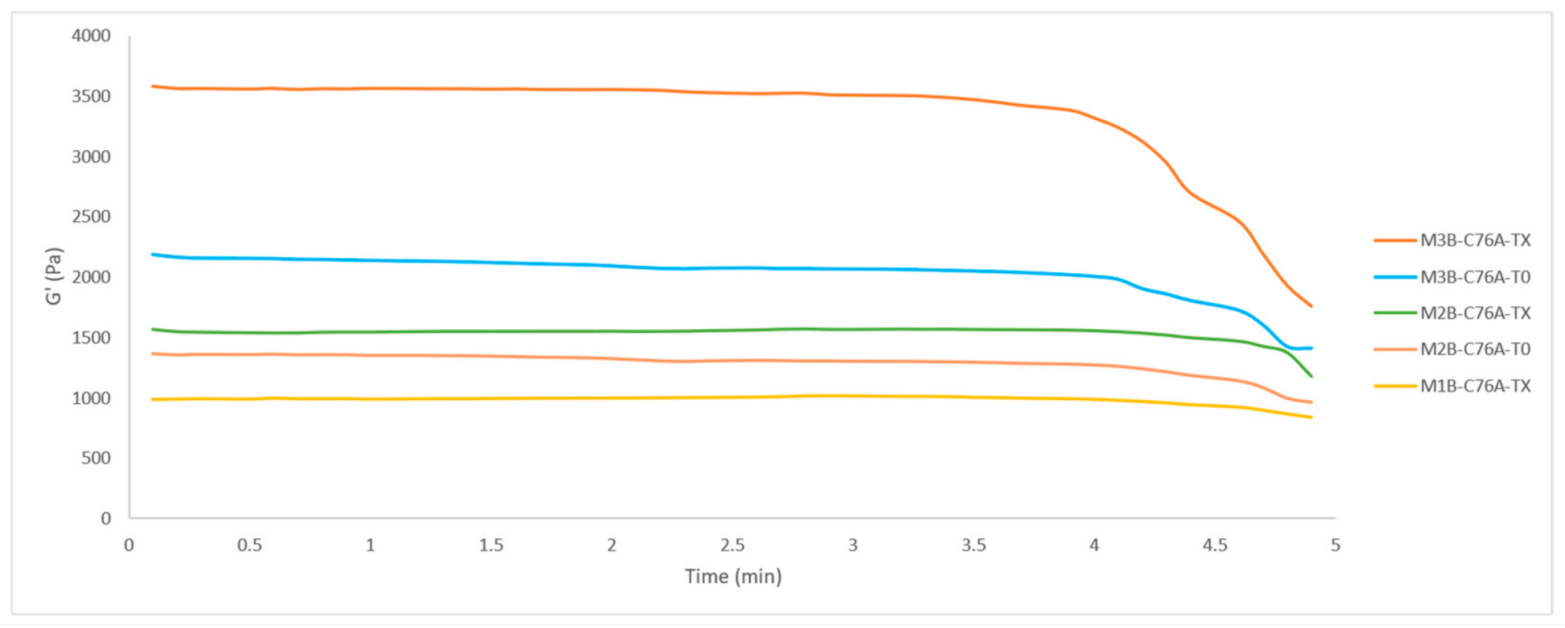
Mean elastic modulus for layers combinations of base membranes with 76% DDA chitosan from Aldrich crosslinked and non-crosslinked.

Figure 11 shows the elasticity of base membranes (C76A) with one layer, two layers with and without crosslinking, and three layers with and without crosslinking for base membranes.

As can be seen, the most elastic membrane was the base tri-layered membrane with crosslinking (M3A-C76A-TX). The following membrane was the base tri-layered membrane without crosslinking. Following this, at around 1500 Pa, base bi-layered membranes with and without crosslinking are found and no significant difference could be observed. Again, the mono-layered membranes were observed last.

Based on the results obtained for C76A, tri-layered crosslinked membranes are more elastic than bi-layered crosslinked membranes and more elastic than mono-layered membranes. The gap between the elastic modulus of tri-layered and two-layered crosslinked membranes is 2.5 times bigger for base membranes and 3.5 times bigger for activated membranes than the gap between their respective tri-layered and bi-layered non-crosslinked membranes. This means that the crosslinking effect between chitosan layers with TPP has a high impact on the elasticity of the membrane and this effect is even more evident in activated tri-layered membranes.

When comparing more than one layer for activated and base membranes with C76A, activated membranes are more elastic than base membranes. This may be due to the lowest thickness of activated membranes and the small pores, which can help to release the internal water content of the membrane. To summarize, the elasticity modulus increases with the number of layers, up to three layers, and with crosslinking.

For chitosan of 85% from Aldrich (C85A), the mean values of the obtained results are shown in Table 8 and Table 9 for activated and base membranes, respectively, until 2 min (elastic modulus is nearly constant).

**Figure 12:**
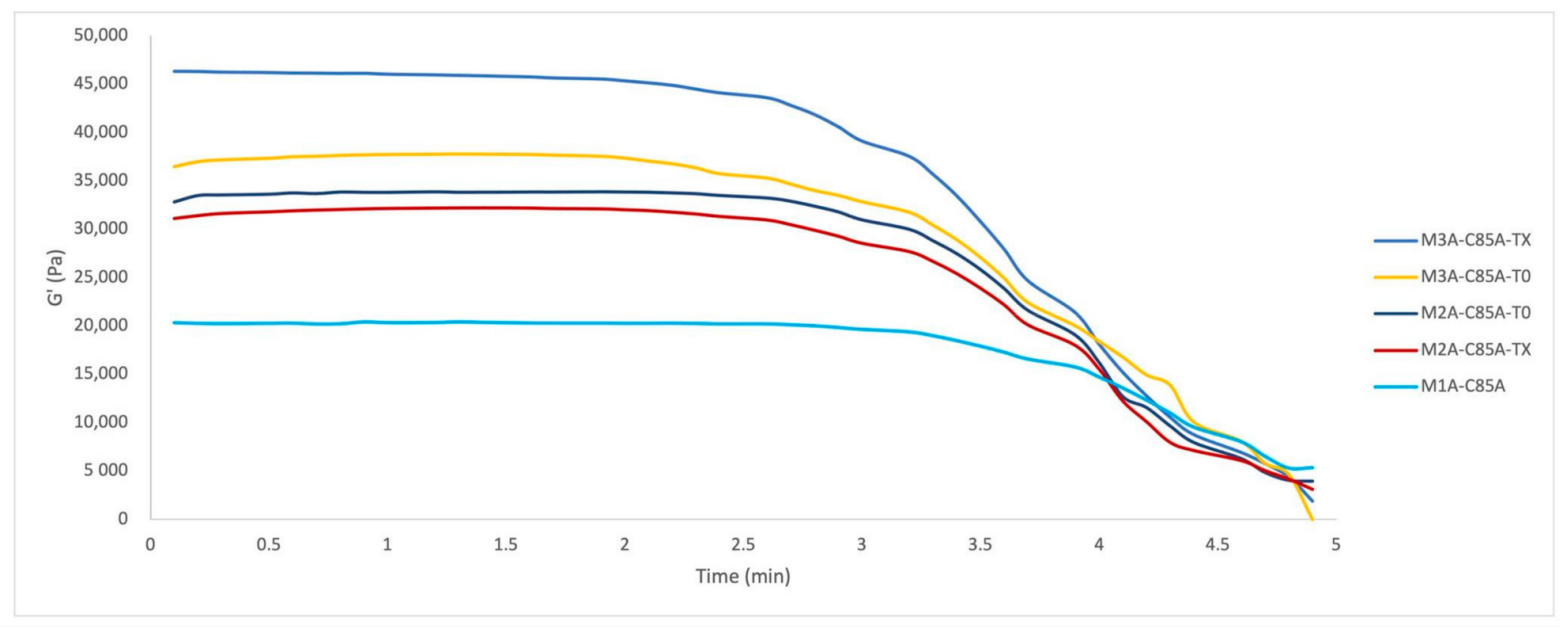
Mean elastic modulus for layer combinations of activated membranes with 85% DDA chitosan from Aldrich crosslinked and non-crosslinked.

As can be seen in Figure 12, the combination with the highest elasticity is the activated tri-layered membrane with crosslinking (M3A-C85A-TX). Following this is the activated tri-layered membrane without crosslinking. Consecutively, the activated bi-layered membranes with and without crosslinking were found, but with no significant difference between them. Finally, the activated mono-layered membrane was the one with the lowest elasticity.

**Figure 13:**
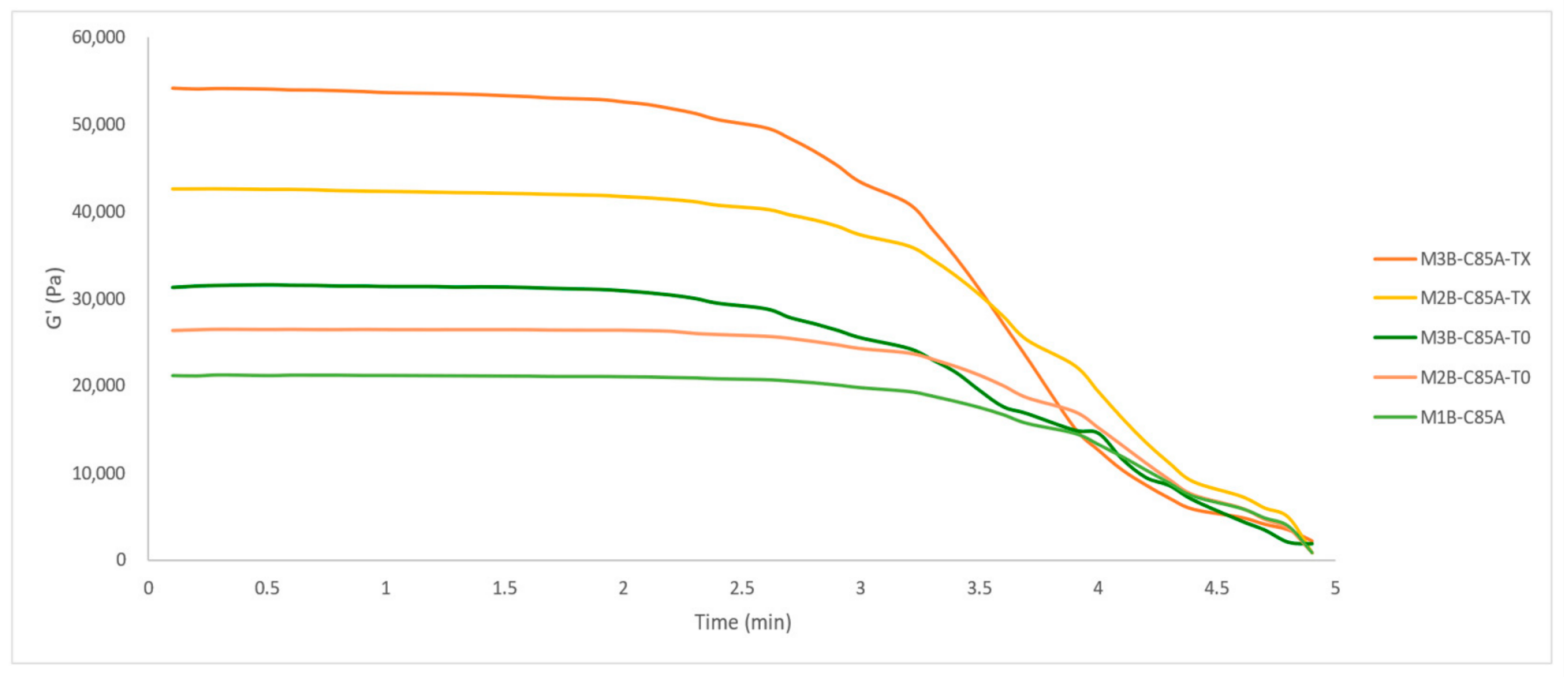
Mean elastic modulus for layer combinations of base membranes with 85% DDA chitosan from Aldrich crosslinked and non-crosslinked.

As can be seen in Figure 13, the combination with the highest elasticity was the base tri-layered membrane with crosslinking (M3B-C85A-TX). Following this is the base bi-layered crosslinked membrane. Consecutively, the base tri-layered membrane without crosslinking was found, followed by the base bi-layered non-crosslinked and mono-layered membranes, as was expected. It can be stated that, for this scenario, the second most elastic membrane is the base bi-layered crosslinked one, instead of the base tri-layered non-crosslinked one, as previously seen. Nevertheless, it needs to be kept in mind that these base membranes with 85% DDA are the most elastic kind. Therefore, here, the effect of crosslinking can be more evident than in the rest of the elastic membranes.

Based on the results obtained for C85A, tri-layered crosslinked membranes were more elastic than bi-layered crosslinked and mono-layer membranes. In this case, the gap between tri- or bi-layered crosslinked membranes is 2.3 times bigger for base membranes and 3.7 times bigger for activated membranes than between their respective tri- or bi-layered non-crosslinked membranes. In brief, as had previously been seen for 76% DDA, for 85% DDA, the elasticity modulus increases with the number of layers, up to three layers, and with crosslinking. Although the crosslinking effect is more evident in activated tri-layered crosslinked membranes, the most elastic membranes are the base tri-layered crosslinked membranes.

In C85A, base crosslinked membranes have more elasticity than activated crosslinked membranes. The explanation of this effect is related to the great elasticity of this kind of membrane, as has already been explained in the previous section. Despite this fact, when elasticity values decay, this effect is not appreciated.

#### 3.3.4. Swelling

As has been previously explained, swelling is a function of DDA. However, the pH also has an influence on the swelling behavior.

When the pH is low, positively charged chitosan exhibits a high swelling index. This is due to the fact that the repulsion force between the positive charges causes greater intermolecular distances and more hydrophilicity [58].

This is the case of chitosan 76% DDA, where the pH of chitosan solution is lower than the pH of 85% DDA, so as to be able to form crosslinking between layers.

As can be observed in Table 10, the values are expressed as means ± standard deviations.

The maximum swell obtained over time for tri-layered crosslinked membranes with chitosan 85% DDA is quite similar for both suppliers.

For chitosan of 76% DDA, apart from having a lower DDA, the tri-layered crosslinked membranes are created with a lower pH of chitosan solution. Therefore, a much higher swell is achieved for these kinds of membranes. When comparing base and activated tri-layered crosslinked membranes, a much higher swell is seen in the activated ones. In this case, we think that this may be due to the DermaStamp^®^ device. This device crushed the membrane, flattening it and causing it to have a wider surface and smaller thickness. This can make the membrane more available for uptaking water.

There is no great difference in swelling between the different chitosan 85% DDA, not even between base and activated tri-layered crosslinked membranes. Notwithstanding this fact, a great difference in swell is found between chitosan of 85 and 76% DDA, being 2.5 times higher for 76% DDA base tri-layered crosslinked membranes and 3.5 times higher for 76% DDA activated tri-layered crosslinked membranes compared to tri-layered crosslinked membranes obtained with 85% DDA.

**Figure 14:**
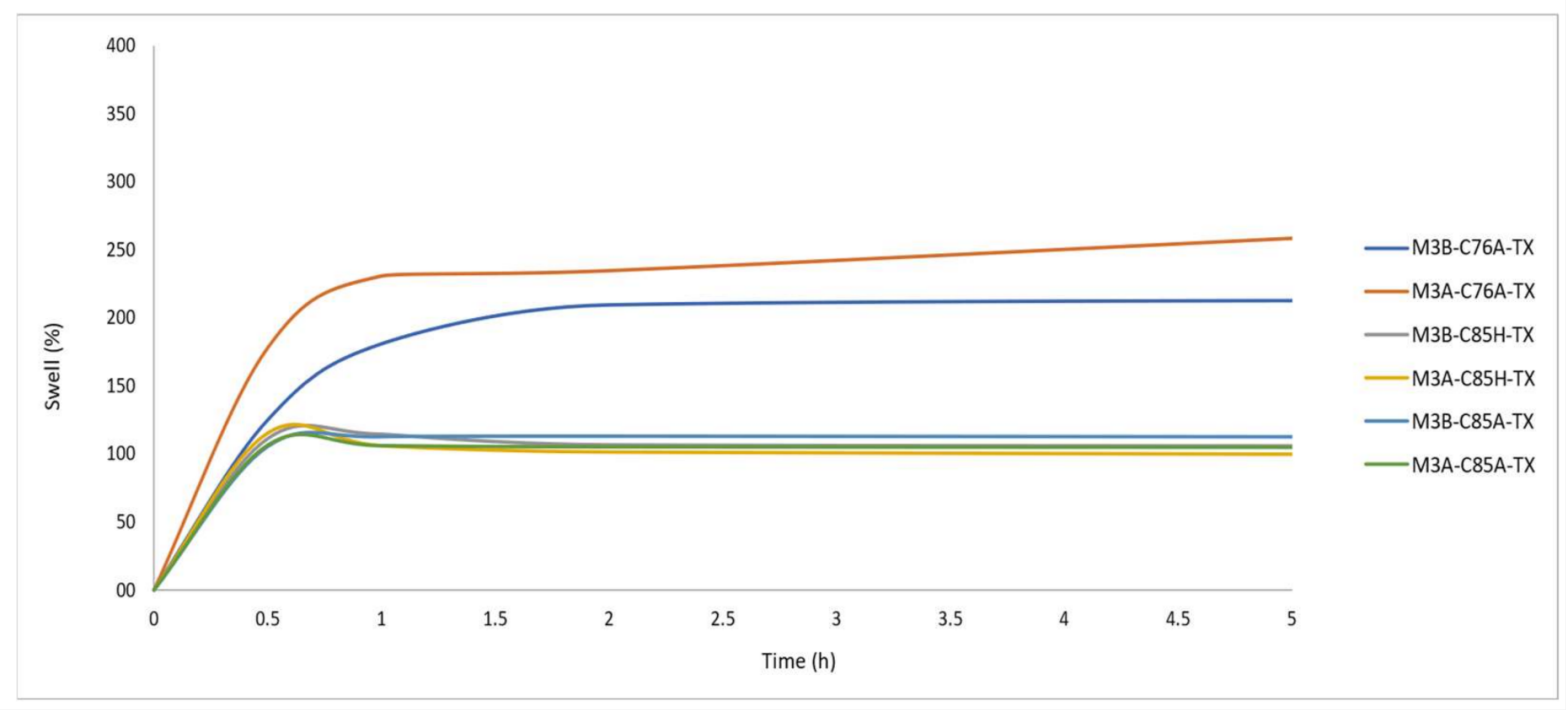
Mean swell kinetics% over time for each kind of tri-layered crosslinked membrane.

When checking the swell over time of all the tri-layered crosslinked membranes, it can be observed in Figure 14 that the highest water absorption is produced in the first 30 min.

The tri-layered crosslinked membranes achieved their maximum swelling at 30 min for 85% DDA chitosan and, for 76% DDA chitosan, at 96 h, as mean values.

## 4. Conclusions

Ionically crosslinked tri-layered membranes were obtained with different kinds of chitosan from different DDA and different natural origins. Reproducible mechanical pores were created in the 85% DDA chitosan crosslinked tri-layered membranes. Different properties could be obtained, depending on the chitosan used.

Chitosan of 85% DDA is suitable for reproducing human skin pores and exhibits a similar elastic pattern to some areas of human skin. It can be seen that chitosan from different origins has some influence on the elastic behavior. Chitosan from Aldrich has a greater elasticity than HMC+. It looks like chitosan from Aldrich is more reproducible in terms of elasticity measurements. Regarding the swelling capacity, both of them produce very similar results, even between base and activated membranes.

Chitosan of 76% DDA can also be suitable for reproducing some kind of elasticity found in several previous publications. It also has the highest swelling capacity, so this could be interesting for testing hydrating topical products versus a placebo. Nonetheless, a major drawback of this membrane is the difficulty found in creating pores.

The developed method shows a good reproducibility between tri-layered crosslinked membranes obtained with the different kinds of chitosan.

It has also been demonstrated that crosslinking and the use of three layers are important issues for increasing the membrane elasticity to get close to the human skin elasticity modulus. As many layers are combined, more elasticity is achieved. In addition, if these layers are crosslinked, the elasticity increases much more. The effect of crosslinking is more evident in activated membranes from both kinds of DDA, although the most elastic membranes are base membranes from C85A.

These membranes are basic scaffolds composed of three layers.

Our future research, in order to improve and increase the number of properties of the membrane related with human skin, will aim to study different combinations of chitosan with several other materials, such as hyaluronic acid, collagen, elastin, and lipids, and they could be added in specific layers for differentiating them as they are naturally found in human skin. Hence, tailor-made layers could be performed and they could represent some aspects of different skin layers, depending on the materials added.

Several active ingredients and topical products could also be tested in these membranes so as to observe their effect on different parameters, such as the elasticity, hydration, water loss, effect on pore reduction, permeation trends, scar treatments, etc.

## Figures and Tables

**Table 1:** Mean tri-layered crosslinked membrane weights of a 2 cm diameter with standard deviation (SD) and mean thickness with standard deviation (SD) obtained for the different chitosan.

Membrane ID	Mean Membrane Weight/g ± SD	Mean Membrane Thickness/mm ± SD
M3B-C85A-TX	0.4 ± 0.03	0.9 ± 0.01
M3A-C85A-TX	0.3 ± 0.03	0.7 ± 0.03
M3B-C85H-TX	0.5 ± 0.1	1.1 ± 0.1
M3A-C85H-TX	0.4 ± 0.1	0.8 ± 0.05
M3B-C76A-TX	1.3 ± 0.5	1.9 ± 0.5
M3A-C76A-TX	0.7 ± 0.1	1.6 ± 0.2

**Table 2:** Mean values and standard deviations (SD) of the pores area µm^2^ of tri-layered crosslinked membranes of 85% DDA. Nº is the number of pores.

Membrane ID	Nº Pores Measured	Mean Pore Area µm^2^	SD of Pore Areas between Membranes µm^2^	SD of Pore Areas in a Membrane µm^2^
M3A-C85H-TX	35	35,879	±2280	±2535
M3A-C85A-TX	35	35,823	±2488	±3092

**Table 3:** Mean water permeation (w perm) and standard deviation (SD) through the base tri-layered crosslinked membrane.

t/min	Mean w perm/g ± SD M3B-C85A-TX	Mean w perm/g ± SD M3B-C85H-TX	Mean w perm/g ± SD M3B-C76H-TX
0	0	0	0
5	0.048 ± 0.1	0.2 ± 0.23	0.07 ± 0.15
10	0.18 ± 0.22	0.46 ± 0.48	0.18 ± 0.38
15	0.40 ± 0.40	0.63 ± 0.66	0.38 ± 0.56
20	0.62 ± 0.59	0.78 ± 0.84	0.52 ± 0.73
25	0.85 ± 0.78	0.903 ± 0.99	0.66 ± 0.92
30	1.05 ± 0.95	1.03 ± 1.16	0.79 ± 1.09
35	1.21 ± 1.12	1.13 ± 1.28	0.88 ± 1.19
40	1.41 ± 1.35	1.24 ± 1.42	0.98 ± 1.29

**Table 4:** Mean water permeation and standard deviation (SD) through the activated tri-layered crosslinked membrane.

t/min	Mean W perm/g ± SD M3A-C85A-TX	Mean W perm/g ± SD M3A-C85H-TX	Mean W perm/g ± SD M3A-C76A-TX
0	0	0	0
1	18.58 ± 0.83	16.37 ± 1.63	0.05 ± 0.10
2	26.76 ± 0.80	25.97 ± 1.29	0.08 ± 0.16
2.5	28.77 ± 0.20	28.66 ± 0.08	0.12 ± 0.18
5	-	-	0.15 ± 0.21
10	-	-	0.43 ± 0.58
15	-	-	0.65 ± 0.87
20	-	-	0.88 ± 1.14
30	-	-	1.32 ± 1.53
40	-	-	1.93 ± 1.72

**Table 5:** Mean and standard deviation (SD) of the elastic modulus of base and activated tri-layered crosslinked membranes until 2 min.

Membrane ID	Mean G’ (Pa) ± SD
M3B-C76A-TX	3359.46 ± 968.97
M3A-C76A-TX	5402.78 ± 1096.34
M3B-C85H-TX	34,378.43 ± 2702.69
M3A-C85H-TX	35,538.68 ± 9561.26
M3B-C85A-TX	53,633.68 ± 2413.52
M3A-C85A-TX	45,997.65 ± 5515.39

**Table 6:** Mean and standard deviation (SD) of the elastic modulus for activated membranes with chitosan 76% DDA from Aldrich crosslinked and non-crosslinked until 2 min.

Membrane ID	Mean G’ (Pa) ± SD
M3A-C76A-TX	5402.78 ± 1096.34
M3A-C76A-T0	2587.29 ± 608.81
M2A-C76A-TX	1678.20 ± 114.91
M2A-C76A-T0	1528.03 ± 23.25
M1A-C76A	874.70 ± 161.95

**Table 7:** Mean and standard deviation (SD) of the elastic modulus for base membranes with chitosan 76% DDA from Aldrich crosslinked and non-crosslinked until 2 min.

Membrane ID	Mean G’ (Pa) ± SD
M3B-C76A-TX	3559.46 ± 968.97
M3B-C76A-T0	2141.58 ± 198.96
M2B-C76A-TX	1547.65 ± 15.44
M2B-C76A-T0	1349.24 ± 102.28
M1B-C76A	996.11 ± 6.48

**Table 8:** The mean and standard deviation (SD) of the elastic modulus for activated membranes with chitosan 85% DDA from Aldrich crosslinked and non-crosslinked until 2 min.

Membrane ID	Mean G’ (Pa) ± SD
M3A-C85A-TX	45,997.65 ± 5515.39
M3A-C85A-T0	37,512.59 ± 4967.79
M2A-C85A-TX	31,954.31 ± 4153.35
M2A-C85A-T0	33,738.43 ± 1584.60
M1A-C85A	20,299.41 ± 1053.55

**Table 9:** Mean and standard deviation (SD) of the elastic modulus for base membranes with chitosan 85% DDA from Aldrich crosslinked and non-crosslinked until 2 min.

Membrane ID	Mean G’ (Pa) ± SD
M3B-C85A-TX	53,633.68 ± 2413.52
M3B-C85A-T0	31,332.35 ± 7033.19
M2B-C85A-TX	42,281.37 ± 3739.43
M2B-C85A-T0	26,396.86 ± 2678.85
M1B-C85A	21,199.80 ± 5058.00

**Table 10:** Mean value and standard deviation (SD) of the maximum swell% observed over time for the different base and activated tri-layered crosslinked membranes.

Membrane ID	Mean Maximum Swell% ± SD
M3B-C85A-TX	107 ± 7.4
M3A-C85A-TX	114 ± 6.2
M3B-C85H-TX	114 ± 5.2
M3A-C85H-TX	115 ± 5.3
M3B-C76A-TX	256 ± 63.6
M3A-C76A-TX	386 ± 122.3

## Data Availability

No new data were created or analyzed in this study. Data sharing is not applicable to this article.
